# Validation of the Mechanical Behavior of an Aeronautical Fixing Turret Produced by a Design for Additive Manufacturing (DfAM)

**DOI:** 10.3390/polym14112177

**Published:** 2022-05-27

**Authors:** Fernando Veiga, Trunal Bhujangrao, Alfredo Suárez, Eider Aldalur, Igor Goenaga, Daniel Gil-Hernandez

**Affiliations:** 1 TECNALIA, Basque Research and Technology Alliance (BRTA), Parque Científico y Tecnológico de Gipuzkoa, 20009 Donostia-San Sebastián, Spain; trunal.bhujangrao@tecnalia.com (T.B.); alfredo.suarez@tecnalia.com (A.S.); eider.aldalur@tecnalia.com (E.A.); igor.goenaga@tecnalia.com (I.G.); daniel.gil@tecnalia.com (D.G.-H.); 2 Department of Engineering, Campus Arrosadía, Public University of Navarre, 31006 Pamplona, Spain

**Keywords:** aeronautical fixture, WAAM, DfAM, additive manufacturing

## Abstract

The design of parts in such critical sectors as the manufacturing of aeronautical parts is awaiting a paradigm shift due to the introduction of additive manufacturing technologies. The manufacture of parts designed by means of the design-oriented additive manufacturing methodology (DfAM) has acquired great relevance in recent years. One of the major gaps in the application of these technologies is the lack of studies on the mechanical behavior of parts manufactured using this methodology. This paper focuses on the manufacture of a turret for the clamping of parts for the aeronautical industry. The design of the lightened turret by means of geometry optimization, the manufacture of the turret in polylactic acid (PLA) and 5XXX series aluminum alloy by means of Wire Arc Additive Manufacturing (WAAM) technology and the analysis by means of finite element analysis (FEA) with its validation by means of a tensile test are presented. The behavior of the part manufactured with both materials is compared. The conclusion allows to establish which are the limitations of the part manufactured in PLA for its orientation to the final application, whose advantages are its lower weight and cost. This paper is novel as it presents a holistic view that covers the process in an integrated way from the design and manufacture to the behaviour of the component in use.

## 1. Introduction

The COVID-19 pandemic was a major setback in the estimated growth of the various manufacturing industries. After the last few years, it seems that the world is finally leaving the pandemic behind, although the risk of new waves will remain. Most industrial sectors have shown signs of continued rapid recovery to pre-pandemic levels, as suggested in [1], although labor market developments are less encouraging, particularly in the case of the civil aeronautics industry. With a Europe at war, this recovery forecast could be further undermined. Moreover, this geopolitical situation is expected to have a direct effect on both the growth of military aerospace spending and the shortage of primary energy and raw materials in some countries [2]. What seems clear is that in this uncertain situation for the industry, current trends and the modernization of manufacturing paradigms are essential. In this direction, three of the lines that receive the most attention are: modular manufacturing [3], sustainable and environmentally friendly manufacturing with less use of resources [4] and portable production systems [5].

The design of flexible clamping systems is a subject that has historically been of great interest to manufacturing engineers because of its impact on the quality of manufactured parts. Added to this is the reality that production demands are becoming increasingly stringent in terms of greater product variety, shorter runs, shorter manufacturing times and higher quality requirements. This inevitably leads the industry to need greater flexibility and automation in its manufacturing process in order to achieve a competitive and profitable product, requiring an increasingly exhaustive study of flexible clamping elements [6,7].

In this respect, it should be noted that the quality and profitability of the manufactured parts depend to a large extent on tooling, and its cost can represent between 10 and 20% of the cost of the manufacturing system, while the use of flexible fixturing can mean a saving of 80% in the cost of tooling [6].

The fixturing is used to locate, clamp and support the part during the manufacturing process, and its behavior affects the outcome of the considered process (machining, welding, assembly, etc.) in terms of qualty, cost and performance. In addition, fixtures have a major impact on the development of processes and on the capabilities of machine tools. They are usually designed as a subsystem and independently from aspects such as cycle times or the cutting process itself [8], although they have an important influence on these aspects, and even fixturing and process can be considered as mutually dependent [9].

Economically, fixtures have a major impact on manufacturing, and there are two types of situations: (1) turnkey developments of the machine/system + fixturing system, and (2) developments for use on existing machines and systems. It is in the latter group where most of the tooling designs are made, reaching 74% of the fixtures [8]. For this reason, it is important to be able to adapt the tooling to existing resources by means of flexible and modular solutions that reduce the economic impact of its development.

Additive design and manufacturing or 3D printing is a manufacturing technology that aligns with these trends. It involves the production of parts by adding materials layer by layer, so it is considered a green production method with minimal or no material waste, this waste sometimes deriving from inaccurate geometry [10]. One of the 3D printing techniques for application in polymers is fused deposition modelling (FDM) [11]. In the use of these technologies, the mechanical properties of the material are due to some process parameters, such as the weft angle and orientation, the deposition rate or the filler density [12]. The FDM production process is a technique that is applied to thermoplastic polymers, such as polycarbonate (PC), polyether ether ketone (PEEK), acrylonitrile-butadiene-styrene (ABS) and, as in this paper, polylactic acid (PLA) [13].

Among the additive manufacturing technologies oriented to the manufacture of metal parts, one of those based on direct energy deposition is Wire Arc Additive Manufacturing [10,14]. It is based on the fusion of materials in the form of wire by means of the action of a heat source produced by the action of an electrical source. The authors of this paper have already presented an application of this technology to the manufacture of topologically optimized aeronautical fixturing by WAAM [15]. Structural optimization aimed at finding an optimal geometry based on a set of constraints is known as topological optimization and is one of the keys to part light weighting in Design for Additive Manufacturing (DfAM). Finite element analysis is used in an iterative manner to determine the minimum amount of material that satisfies the requirements set by the constraints [16]. Table 1 summarizes the previous related work conducted by this research group and the aim and motivation of the current paper.

The novelty of this paper lies in the integration of Additive Manufacturing (AM), both in polymers (FDM) and metals (WAAM), among the manufacturing methods of tooling components, by means of: the analysis and selection of the components whose additive manufacturing presents clear advantages in terms of performance, functionalities, costs and manufacturing times; the selection of the appropriate materials and AM processes; the selection of the appropriate commercial systems for the AM of tooling components; the definition of “Design for Additive Manufacturing” techniques; and finally the validation of the mechanical behavior of the manufactured component either by means of finite element analysis (FEA) and by mechanical characterization in a laboratory test bench.

## 2. Materials and Methods

### 2.1. Case Study: Fixing Turret

**Figure 1:**
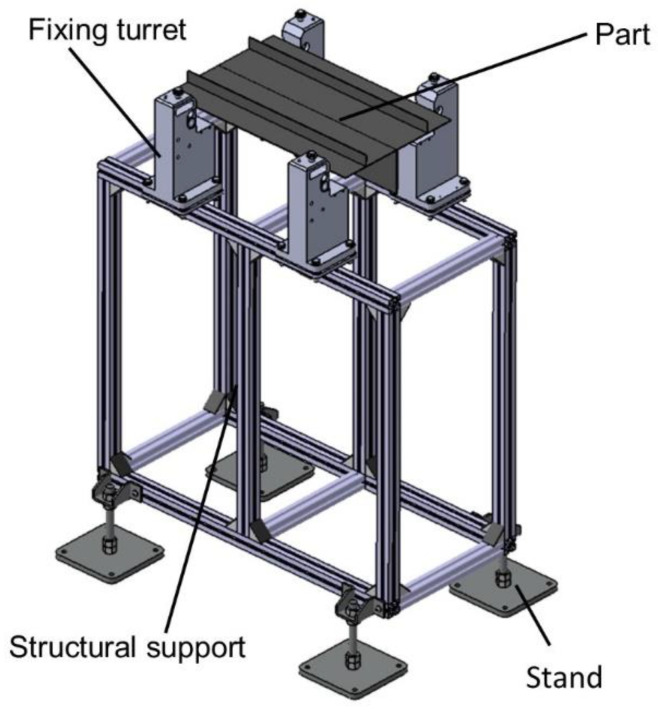
Location of the fixturing turrets for the anchoring of aeronautical parts (Adapted from [15]).

The case study chosen for the application of redesign and 3D printing techniques was that of a turret for fixing aeronautical parts. The turret is mounted on a metal structure of extruded profiles with a linear guide for adjusting the distance between support points. The complexity of the turret is intermediate. The purpose of the work carried out once the part is fixed is to support the assembly and disassembly of aeronautical components. Figure 1 shows an example of the assembly with the four support points in the case of a standard part. These support points may vary depending on the length of the part. This case study was previously presented by the authors in [15], although with a different redesign approach and without carrying out the experimental validation of the mechanical behavior in the use of the redesigned turret.

Analyzing the assembly, the metallic structure formed by extruded and welded bars is of low complexity and low cost, as well as not being complex to manufacture. This structure could even be assembled in situ at the site where the clamping fixture needs to be deployed. The linear carriages and plates for clamping the turrets are mainly commercial rails. In the case of the turret, the tolerances are tighter and their manufacture more complex involving the roughing and finishing of machining processes. Therefore, the aim of this paper was to evaluate the suitability of the clamping turret for 3D printing.

### 2.2. Design for Additive Manufacturing (DfAM) Methodology

The turrets, shown in the previous section, are more complex elements that have direct contact with the aeronautical part that hold it and provide access to certain areas and avoid collisions in assembly work and auxiliary tasks. They are elements screwed to the plates in the structure with adjustable distance to adapt to different types of parts. Their manufacture is the costliest because it involves the finishing of machining operations to meet tolerances, so it is clear that the 3D printing of these components offers advantages over conventional manufacturing processes. The main advantages are that their manufacture can be carried out in the manufacturing plant, even in circumstances of on-board production, and the use of material for manufacture is optimized by reducing the volume of material required to build the part. This provides a more flexible and lighter solution.

**Figure 2:**
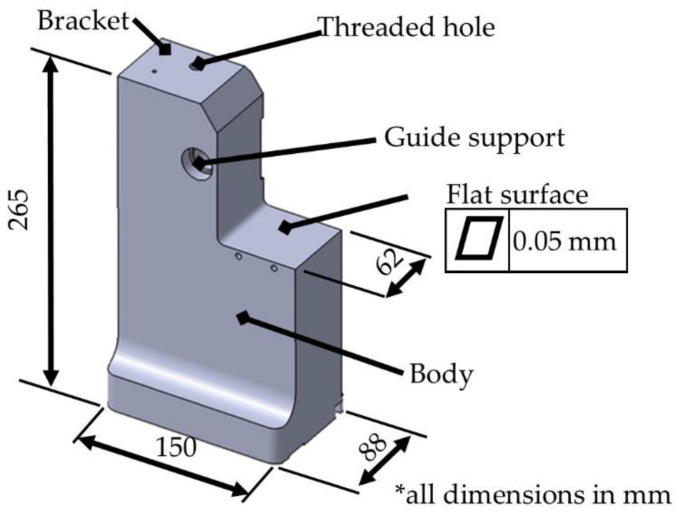
Location of the fixturing turrets for the anchoring of aeronautical parts.

Figure 2 shows the main dimensions of the part, which is currently manufactured in 5XXX series aluminum alloy (AA5xxx). It has a weight of 6 kg. The top surface has to accommodate a threaded hole and the body. In addition, there is another horizontal platform to support the workpiece, which must have a demanding flatness.

The DFAM methodology starts with the definition of a structural optimized part based on arcs and hollow volumes using Dassault Systemes Catia software, ensuring that the functional requirements of the part are met. For the manufacture of the turret, the deposition source trajectories are defined using an Autodesk Powermill Additive CAM for the 3D printing of the part using WAAM and FDM technology.

### 2.3. Additive Manufacturing: Material, Machine and Parameters

For the 3D printing of the parts, two deposition technologies were used: FDM technology for polymer-oriented parts and WAAM technology based on Gas Metal Arc Welding (GMAW) for use on metals.

FDM builds three-dimensional parts by melting and advancing a thin plastic wire through a computer-controlled extrusion head, producing thermoplastics parts. These parts generally have a greater geometric accuracy than those produced by direct wire deposition technologies, such as WAAM. In this case, the turrets were printed in PLA. The PLA 3D printing material offers strength and heat resistance ideal for low-cost, quick rapid prototyping in a wide range of colors. FDM technology is one of the technologies that allows the manufacture of parts with relatively complex geometries, with cavities or double curvature walls. The printer used for the PLA turret manufacturing was the Ultimaker S5, with a printable volume of 330 × 240 × 300 mm, and compatible to print high-strength carbon fiber and glass filaments. The layer resolution was down to 20 microns with any 2.85 mm diameter of raw material.

**Figure 3:**
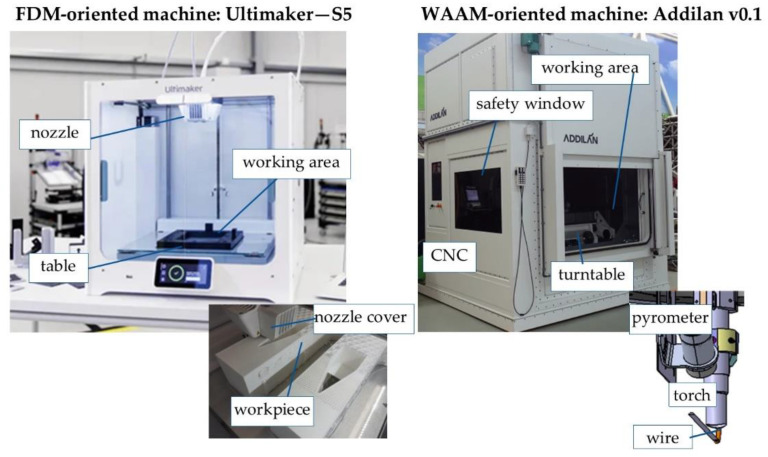
Machines and systems used for the manufacture of the part in FDM (Ultimaker S5) and WAAM (Addilan v0.1).

The WAAM technology with GMAW system has the main advantage of being able to work at high deposition rates with a synergic control system of the welding arc. The main disadvantage in this case lies in the impossibility of making certain geometries as the molten material is not stable in its liquid phase and can cause spattering. In the case of the WAAM technology, an Addilan v0.1 machine with a Titan XQ 400 AC puls (EWM) welding equipment fed the GMAW welding torch, which was placed on the Fanuc Arc Mate 100-iC robot arm in order to produce the parts layer by layer. This setup was also equipped with the M drive 4 Rob5 XR RE (EWM) wire feeding equipment and the shielding gas system. Figure 3 below shows the machines and systems used for the 3D printing of the turrets.

The parameters employed for 3D printing in both technologies are listed in Table 2. It summarizes the parameters achieved after tuning the technologies for the correct regime deposition of the target materials. Part of this work was presented by the authors in [18].

### 2.4. Mechanical Characterization

**Figure 4:**
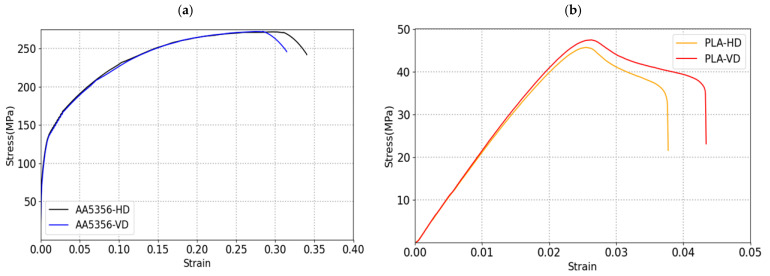
Engineering stress–strain curves of (**a**) AA5356 and (**b**) PLA in the HD and VD directions.

Tensile specimens with the dimensions of 4 mm in diameter and 22 mm in gauge length (ASTM E8 standard) were extracted in the horizontal direction (HD) and in the vertical direction (VD) for WAAM AA5356 material and for the PLA; the dimension of the specimen was based on ASTM D638 standards. Tensile tests were carried out for PLA at speed of 5 mm/min with a load cell of 10 kN and for WAAM AA5356 alloy using a 100 kN load cell at speed of 1 mm/min using Intron testing equipment. Several tests were carried out on both materials to study the mechanical behavior in different directions. The stress vs. displacement curves of WAAM AA5356 and PLA were plotted and shown in Figure 4. The strain–hardening behavior was tested, and it increased continuously after yielding in both cases, whereas a sudden drop in yield stress followed by a steady state was observed in the samples. The elongation of PLA was almost 40%, whereas that of the AA5356 alloy was almost 80%.

A detailed comparison of the tensile test results of the WAAM AA5356 alloy and the PLA is given in Table 3. From Table 3, it is concluded that the mechanical properties appear to be almost similar for the HD and VD directions.

### 2.5. Mechanical Behaviour Testing

**Figure 5:**
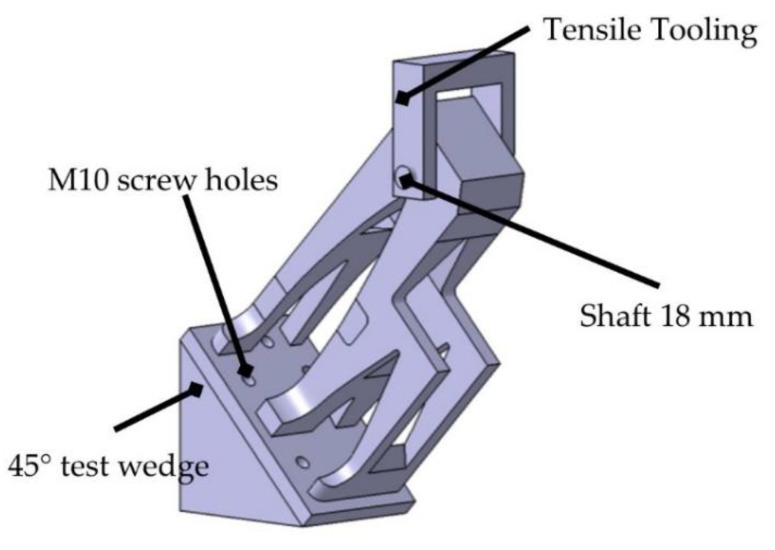
Turret tensile test configuration: 45° test case.

Tensile and compression tests were carried out on the parts in order to verify their mechanical behavior. For this purpose, a simple fixture was designed that allows the part to be clamped. The load was applied at two angles, 0° and 45°, as shown in Figure 5.

In order to be able to attach and apply the intended loads, a series of holes were drilled in the upper part and at the base of the turrets. In the upper part, an 18.5 mm transverse hole was drilled in order to be able to fit the pulling tool. On the other hand, the lower part was fastened by means of five M10 bolts that allow the part to be fastened to the test bench flange. In the case of the 45° test, it was also necessary to manufacture a shim that allows the part to be oriented in this way inside the test bench.

The force range to be applied during the test was determined according to the maximum allowable deformation of the part during operation. For this purpose, a control point was defined on the front face of the turret. The maximum admissible displacement at this point was ±0.15 mm.

## 3. Results

### 3.1. Design for Additive Manufacturing (DfAM) Methodology

**Figure 6:**
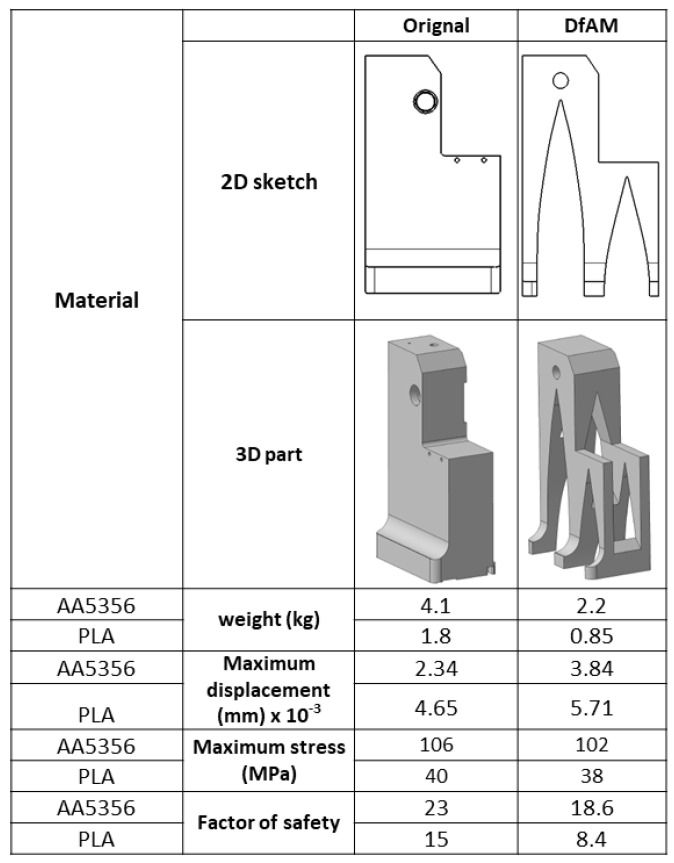
Comparison of results from the original and DfAM parts for PLA and WAAM AA5356.

The DfAM method of topology optimization is a type of structural optimization technique that can optimize the material arrangement within a given design space. However, it should be noted that, during the topology optimization process, some manufacturing constraints, such as the minimum feature size, must also be taken into account. Since topology optimization can help designers to obtain an optimal complex geometry for additive manufacturing, this technique can be used to optimize the turret parts in this article. The comparison between the original part and the DfAM part of different material based on weight reduction and design complexity is explained here, as shown in Figure 6 for the turret. Inspired by the arch design, the 2D scheme was drawn and converted into 3D parts.

To evaluate the applicability of the given parts, some physical properties of the parts were compared. For example, the weight of the parts was reduced by almost 25–30% in both materials, the elastic limits were almost similar and the safety factor decreased. Finally, it is concluded that, from a design point of view, this change in physical properties is acceptable for the DfAM of parts using both materials.

### 3.2. Additive Manufacturing of the Fixing Turret

The manufacturing process was carried out in both technologies with matrix manufacturing. This strategy is particularly suitable for the WAAM technology, as the waiting time between layers is used to manufacture the next part. This strategy has already been used by the authors of this paper in 2021 [19]. The objective is to achieve a “quasi”-continuous process, having stops for arc start-up and process maintenance times. Table 4 summarizes process times, deposition rates and the total part weight.

**Figure 7:**
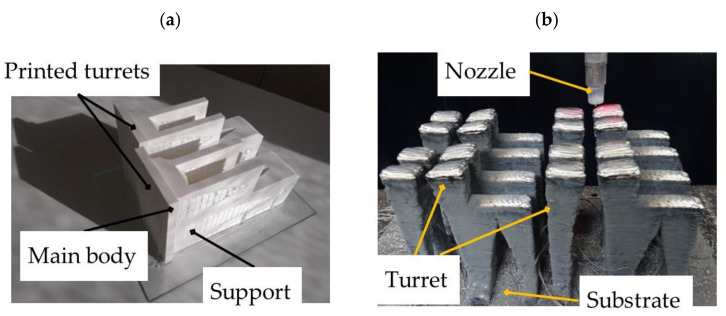
Manufacturing process of the turrets with (**a**) FDM and (**b**) WAAM technology.

In the case of the aluminum part, a 2 × 2 matrix was produced, while for the PLA, two units were printed simultaneously. Figure 7 shows the production in both processes.

Finally, machining processes were carried out on the reference surfaces in the case of the turret manufactured by WAAM. In the FDM-printed parts, the material was removed from the supports to obtain the final part geometry that best fits the theoretical CAD model.

### 3.3. Finite Element Analysis (FEA) of the Mechanical Behavior of the Fixing Turret

**Figure 8:**
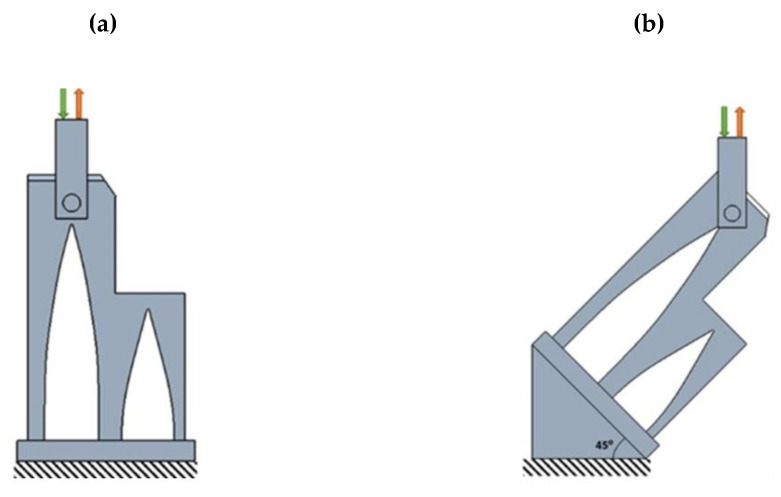
Configuration of the turret, where the tensile and compression loads were applied at (**a**) 0° and (**b**) 45°.

Despite the anisotropy of the material in additive manufacturing processes, an isotropic plasticity model was adopted for both materials. With the aim of evaluating the applicability of the constitutive model for WAAM parts, finite element models of the turret part were built and benchmarked against the experimental results, as given in Section 2.5. All the finite element simulations were conducted in MSC NASTRAN (2020) using solid elements for both PLA and AA5356. The FEM (nonlinear static) simulation was performed by applying the load of 0–500 kg at the control point of each part at the different orientations of 0° and at 45° and the deformation was obtained as output. The configuration of the FEM simulation of the turret is given in Figure 8.

**Figure 9:**
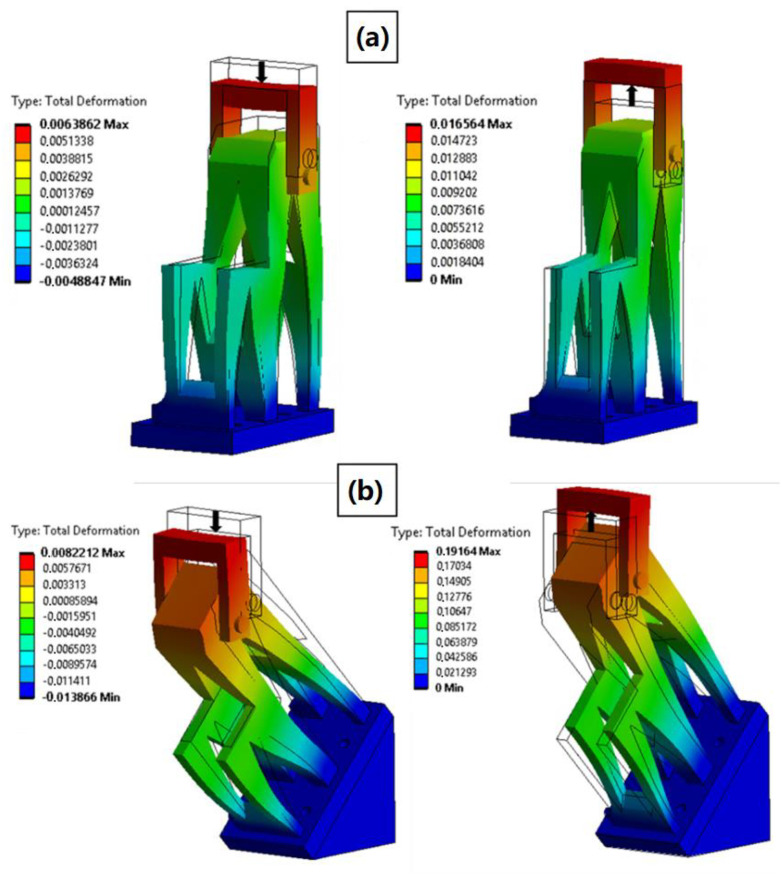
FEM simulation of the turret. Deformation analysis at (**a**) 0° and (**b**) 45° for WAAM AA5356.

The boundary conditions for each part were fixed based on the load applied in the actual application, the bottom surface was fixed in all directions and the static load was applied on the top of the turret surface, as shown in Figure 8. The eight-node linear brick, reduced integration element (C3D8R) was selected for all the parts. We chose the 3D solid elements. The overall CPU time was approximately 40 min for the model. In general, the finite element simulation results show the displacement of the turret part when the load was applied at 0° under a higher tensile loading compared to the compression loading, as shown in Figure 9a. For the load applied at 45°, the deformation is much higher under both tensile compressions compared to the 0° orientation. Figure 9 shows the total displacement of the WAAM AA5356 alloy, when a 500 kg load was applied in tensile and compression tests at different angles. 

**Figure 10:**
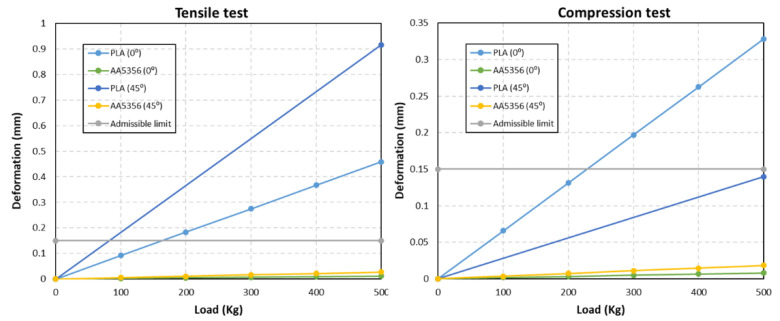
Deformation of the turret for WAAM AA5356 and PLA at different loading weights under the tensile and compression tests.

The complete deformation analysis of the FEM simulation is shown in Figure 10. It can be seen that, under the tensile and compressive testing of the part AA5356 under different loading conditions of 0–500 kg, the deformation of the part shows that it does not exceed the allowable limit. For PLA, in a tensile test, as the load increases, the part undergoes a significant deformation. At 0°, the PLA part shows a higher deformation compared to that at 45°, whereas in the compression test, the PLA part shows less deformation.

## 4. Discussion

### Validation of Mechanical Testing: Comparison of the PLA and WAAM-AA5356 Parts

**Figure 11:**
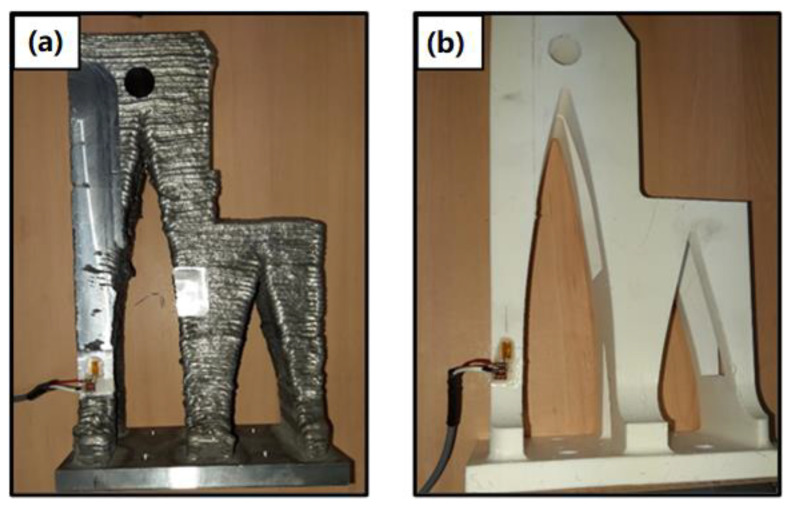
The strain gauge is placed on one of the legs of the parts to be tested: (**a**) WAAM AA53536 and (**b**) PLA parts.

After performing the FEM simulation on the given turrets of two different materials, mechanical tests were performed in order to compare the mechanical behavior of the parts. The mechanical testing procedure and its setup are described in Section 2.4. After manufacturing the parts, the necessary surface of the parts was machined; the measuring sensors (strain gauges) were placed on the PLA and aluminum parts in order to measure the deformations suffered when subjected to compression and tensile forces. First, a strain gauge was placed on one of the legs of the parts, as shown in Figure 11.

**Figure 12:**
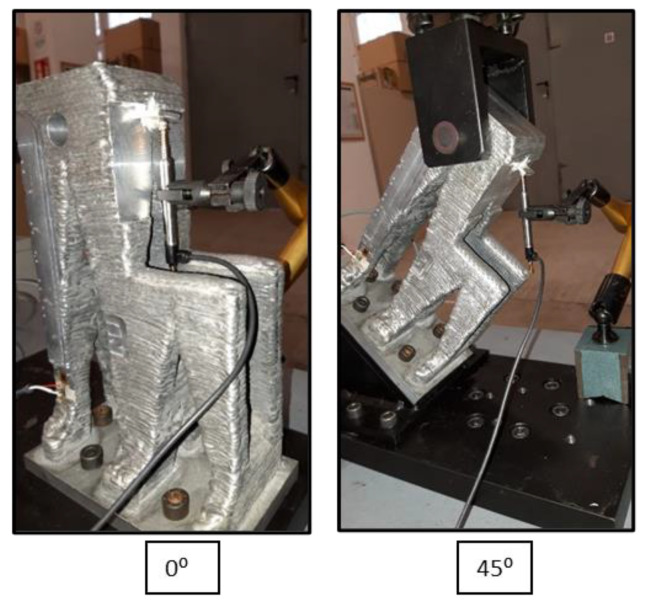
Positioning of the LVDT sensor in the test for WAAM AA5356 oriented at 0° and 45°.

In addition, a metal bracket was added to the front of the parts to measure the displacement of the control point by means of an LVDT, as shown in Figure 12.

**Figure 13:**
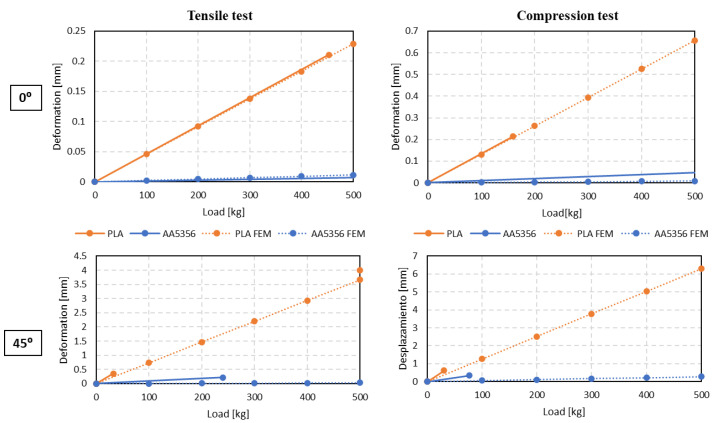
Deformation at different loadings under tensile and compression tests compared with the FEM results oriented at 0° and 45° on PLA and AA5356 turrets.

Once the sensors were placed, the tests were carried out oriented at 0° and 45° with the part fastened at the bottom by means of screws and at the top by means of a tensile fixture, as shown in Figure 12. These tensile as well as compression tests were carried out with the turrets. Figure 13 shows the results of the displacement of the control point measured by the LVDT in the tensile and compression tests for the two orientations in the turrets. In addition, the values obtained were compared with those of the reference limit.

It is clear from the graph that, at 0° deformation under tensile and compression tests, both materials are close to the admissible limit. At 45°, deformation is larger in both materials. So, it is concluded that the parts built from additive manufacturing go under a higher deformation when the load is applied at 45°, which is due to the anisotropy of the material in the different directions, and it is clearly captured by the FEM simulation results.

This comparison between the FEM and experimental tests shows that FEM model allow us to understand the behavior of the mechanical properties of different materials. This kind of validation helps the designer to choose the different additive manufacturing technology and material based on different applications. Similarly, there are several research papers that have studied the DfAM parts and performed various type of experimental validation tests in order to compare the mechanical properties of the parts. e.g., [20] designed and manufactured an aerospace bracket using additive manufacturing; [21,22] manufactured lightweight spacecraft components; and [23] experimentally investigated 3D-printed material properties based on SLA and SLM. The current manufacturing solution made lighter-weighted parts, which is also achieved by Veiga et al. [15] and Suárez et al. [17]. By carrying out these tests and manufacturing the parts, as well as adopting a new design, we achieved a maximum holistic view of the manufacture of different complex parts. 

## 5. Conclusions

This paper presented a methodology for the design of a part using 3D printing technologies. The technologies used were FDM and WAAM. Their mechanical behavior was simulated and validated by means of an experimental bench test. Some of the conclusions that can be drawn are:

- Initially, the material manufactured by AM was characterized by means of specimens designed for this purpose. The mechanical properties reported for the PLA manufactured by FDM are YS 43 MPa, UTS 47 MPa and elongation of 41% of mean; for the case of AA5356 manufactured by WAAM, they are YS 147 MPa, UTS 270 MPa and elongation of 79% of mean with a higher anisotropy in the results.
- A design solution based on arches was adopted, a solution aimed at lightening the parts. This design was applied to an aeronautical turret. The reasoning behind the design is inspired by civil structures. The result is a part that is approximately 50% lighter than the original part.
- It was manufactured with a matrix strategy to optimize manufacturing times, especially in WAAM. This technology is faster in the manufacture of the part with less geometric accuracy.
- The mechanical behavior of the parts manufactured by PLA and WAAM was simulated and validated with experimental tests. It was observed that the model is capable of estimating the observable deformations in the real part.
- The mechanical behavior of the part is better in metal fabrication with WAAM in aluminum for its mechanical properties, although the polymeric solution in PLA by FDM could be sufficient in some application cases and for parts where light weighting is a critical constraint.

## Figures and Tables

**Table 1:** Summary of the related work carried out by the research group.

References	Suarez et al. [17]	Veiga et al. [15]	Current Work
**Aim**	The paper aims to lighten three different parts of the fixturing system by means of DfAM techniques for Additive Manifacturing (AM) with topological optimization, in different materials (polymers and metals).	This article focuses on Ref A of the previous work, which is the one used in the current paper, aiming at the actual fabrication of the part by WAAM.	This article aims to complement those previously presented. Considering and observing the limitations of the WAAM for topological optimization, a vault structure design to lighten the volume of the part is adopted. The part is manufactured using metal and polymer material. Its mechanical behavior is tested and compared with the FEM model.
**Procedure**	Commercial software based on polyNURBS was applied to topologically optimize the AM parts, and thethe topologically optimised solution was adapted. The mechanical behavior of the parts was analysed using finite element methods (FEM).	The topological optimization solution chosen in the previous work was adapted considering the constraints of the WAAM technology and by means of the characterization of the materials. The part was manufactured using different metals.	Inspired by civil engineering applications, we opted for a design in the form of arches made by hand. This type of design, more suitable for certain AM technologies, should be automated in future steps. The incorporation of polymer manufacturing allows for a more accurate part in terms of overall geometry after printing and a part with maximum lightness.
**Design Solution**	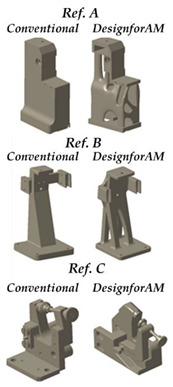	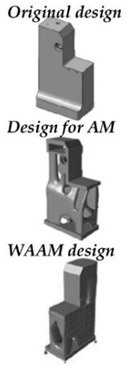	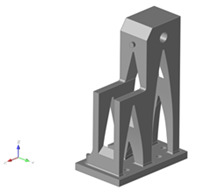
**Achievements**	The current manufacturing solution was made lighter, the mechanical behavior of the parts was tested under different materials and load conditions. Finally, a methodology based on artificial neural networks (ANN) was developed to interpolate in other working conditions.	A methodology for the design and fabrication of an optimized and subsequently corrected real part was carried out taking into account the limitations of WAAM in several of the main weldable materials.	By carrying out these tests and manufacturing the parts, as well as adopting a new design aimed at maximum light weighting, a holistic view of the manufacture of parts of medium size and complexity is given.

**Table 2:** Process parameters for FDM and WAAM.

Material	Layer Height	Wall Thickness	Filling Pattern	Filling Density	Support Pattern	Support Density	
PLA	0.15 mm	1.2 mm	Triangles	100%	Triangles	10%	
**Material**	**Wire Diameter (mm)**	**Transfer Mode**	**Wire Feed Speed WFS (m/min)**	**Travel Speed (cm/min)**	**Layer Height (mm)**	**Current I (A)**	**Voltage V (V)**
AA5356	1.2	Pulsed AC	8	168	1.5	128.36	16.61

**Table 3:** Statistical analysis on the tensile results of AA5356 alloy and PLA.

		AA5356	PLA
0.2% Yield strength (MPa)	HD	148.25 ± 5	42.4 ± 0.34
	VD	146.88 ± 4	43.5 ± 0.89
UTS (MPa)	HD	276.67 ± 3	46.42 ± 0.45
	VD	264.33 ± 4	48.68 ± 0.41
Elongation (%)	HD	81.58 ± 4	39.32 ± 0.09
	VD	78.60 ± 3	42.20 ± 1.02

**Table 4:** Statistical analysis on the tensile results of the AA5356 alloy and PLA.

Material	Production Time (h/part)	Deposition Rate (kg/h)	Printed Part Weight (kg/part)
AA 5356	5	1.44	2.22
PLA	103	0.01	0.85

## Data Availability

Data are available upon request from the corresponding author.
